# Assessment of the psychosocial and economic impact according to sex in non-small cell lung cancer patients: an exploratory longitudinal study

**DOI:** 10.1186/s40359-020-00489-z

**Published:** 2020-11-23

**Authors:** N. Nuria Viñolas, Rosario Garcia-Campelo, Margarita Majem, Enric Carcereny, Dolores Isla, José Luis Gonzalez-Larriba, Juan Coves, Javier De-Castro, Manuel Domine, Piar Lianes, Angel Artal, Jordi Remon, Enriqueta Felip, Pilar Garrido

**Affiliations:** 1 Medical Oncology Department, Hospital Clínic i Provincial de Barcelona, Carrer Villarroel, 170, 08036 Barcelona, Spain; 2 Translational Genomics and Targeted Therapeutics in Solid Tumors, Agusti Pi i Sunyer Biomedical Research Institute (IDIBAPS), Barcelona, Spain; 3 Medical Oncology Department, Complexo Hospitalario Universitario A Coruña (CHUAC), A Coruña, Spain; 4 Medical Oncology Department, Instituto H. Santa Creu i Sant Pau, Barcelona, Spain; 5 Medical Oncology Department, Hospital Universitari Germans Trias I Pujol, Badalona, Barcelona, Spain; 6 Medical Oncology Department, Hospital Lozano Blesa, Zaragoza, Spain; 7 Medical Oncology Department, Hospital Clínico San Carlos, Madrid, Spain; 8 Medical Oncology Department, Hospital Son Llatzer, Palma de Mallorca, Spain; 9 Medical Oncology Department, Hospital Universitario La Paz, Madrid, Spain; 10 Medical Oncology Department, Fundación Jiménez Díaz, Madrid, Spain; 11 Medical Oncology Department, Hospital de Mataró, Barcelona, Spain; 12 Medical Oncology Department, Hospital Miguel Servet, Zaragoza, Spain; 13 Medical Oncology Department, CIOCC Barcelona – HM Delfos, Barcelona, Spain; 14 Medical Oncology Department, Hospital Universitario Vall d’Hebrón, Barcelona, Spain; 15 Medical Oncology Department, Hospital Universitario Ramón y Cajal, Madrid, Spain

**Keywords:** Carcinoma, Non-small-cell lung, Caregivers, Sex characteristics

## Abstract

**Background:**

Little is known about the impact of sex on lung cancer patients from the psychological, economic and social perspectives. This study was designed to explore the psychosocial and economic impact according to sex of metastatic non-small cell lung cancer (mNSCLC) in patients and caregivers.

**Methods:**

Exploratory study of two cohorts of patients starting first-line treatment for mNSCLC. The following questionnaires were administered at baseline, 4 months later and following the first and second disease progression: APGAR, relationship impact scale, DUKE-UNC scale, economic impact in patients and caregiver, and Zarit scale. It was planned to include 1250 patients to get an 80% possibility of detecting as significant (*p* < 0.05) effect sizes less than 0.19 between men and women. Univariate comparisons were made between the tests applied to men and women. Overall survival was estimated with Kaplan–Meier method. Cox analyses were done to estimate hazard ratios (HRs) with 95% CI.

**Results:**

333 patients were included. Most families reported to continue being functional despite the lung cancer diagnosis. Regardless of sex, they did not perceive changes in their partner relationship. Most patients felt their social support was normal. Roughly 25% of people reported a worsening in their economic situation, without remarkable differences by sex. Statistically significant differences were found between both groups regarding the caregiver’s relationship to the patient (more parents were the caregiver in females than in males, *p* < 0.0001) and the caregiver’s employment situation (more employed caregivers in females) (*p* < 0.0001). Most caregivers of both sexes considered that taking care of their relative did not pose a significant burden.

**Conclusions:**

This study provides a preliminary insight into sex-related characteristics in the management of advanced NSCLC and its impact on the emotional, social and economic burden of patients and their caregivers, and recall the high priority of researching in cancer from a sex perspective. Nevertheless, due to the low recruitment rate and the relevant loss of patients during the follow-up, it was difficult to find differences by sex.

**Trial registration:**

ClinicalTrials.gov identifier: NCT02336061.

**Ethics committee:**

Comité Ético de Investigación Clínica del Hospital Clínic de Barcelona, Spain. Reference number: HCB/2014/0705.

## Background

Following the GLOBOCAN 2018 estimates of cancer worldwide incidence and mortality, in both sexes combined, lung cancer is the most commonly diagnosed cancer (11.6% of the total cases) and the leading cause of cancer death (18.4% of the total cancer deaths) globally and among males, and the second in females [1]. According to data from the Spanish Statistics National Institute (INE), in 2017 there were a total of 22,089 (17,241 men and 4848 women) deaths in Spain due to lung cancer [2], which corresponds to a mortality rate of 71.38/100,000 inhabitants (125.56/100,000 in men and 28.39/100,000 inhabitants in women) [3].

About 80% of lung cancers are non-small cell lung cancer (NSCLC) and around 40% of patients have metastatic disease at the time of diagnosis [4].

Historically, lung cancer has been viewed as a male disease, but during the past years there has been a dramatic increase in the incidence in women, attributed to a significant increase in tobacco consumption; indeed, according to the last inform from the Spanish Association Against Cancer [5], the percentage of smoking women is approaching to that in men (18% vs. 27%). As the incidence of lung cancer in women has increased, significant sex-based differences in epidemiology, biology, and treatment responses have become evident [6, 7]. In spite of it, little is known about the impact of sex in lung cancer patients from the psychological, economic and social perspectives [8].

Cancer research and clinical trials have historically focused on efficacy parameters, toxicity and quality of life. However, there is an increasing interest to evaluating the disease’s impact from other perspectives such as caregivers, family functionality and finances, both in the patients and their family setting.

At the time of disease progression, the burden of symptoms and the problems related to social and caregivers support become more evident. Similarly, the economic resources can be also influenced [9]. It is expected that some patients become more dependent, require more resources and time from caregivers. Eventually, caregivers have to reduce their working hours or seek for additional help. The caregiver role in Spain has traditionally assigned to family, usually women [10] but now that lung cancer is no longer considered a men’s disease, little is known about the influence of sex´s patients from the caregivers’ perspective. Around 20–50% caregivers suffer from stress, including changes in their usual routines, changes in family roles, personal health conditions, and occupational and financial disruption [11].

Lung cancer seems to have a greater economic impact than other tumors, due to its poor prognosis and the significance of its symptoms, which can lead to incapacity for the patient (and occasionally the caregiver) in continuing to perform their job role [12]. Most caregivers of patients with NSCLC suffer financial and social harm from the patient’s diagnosis as their social and leisure activities are reduced and they are forced to reduce their working hours [13]. The economic costs associated with the disease are due to the large number of symptoms presented by these patients and their severity, increasing with the progression of the disease and deterioration in the quality of life. There are no data published to date on the overall cost of the disease in Spain.

The present study aimed to explore the differences in the influence lung cancer has on women compared to that on men in terms of social, family and economic impact.

## Methods

### Trial design

This study is a multicenter, prospective, observational, epidemiological, follow-up exploratory study of two cohorts of patients with metastatic NSCLC (male and female), carried out by the Spanish Association for Lung Cancer Research in Women (ICAPEM).

### Patient selection

Eligible patients were ≥ 18 years old, had cytological or histologically confirmed metastatic NSCLC, an Eastern Cooperative Oncology Group performance status (ECOG PS) ≤ 3 and no previous systemic treatment for metastatic NSCLC.

Patients meeting all selection criteria who were going to start first-line treatment for metastatic NSCLC were included in the study. The treatment chosen during the study depended on the oncologist’s judgment. No treatment recommendations or restrictions were stated in the study protocol.

### Evaluations during the study

Information on the demographic variables, medical history, social and economic impact was collected. All the patients’ clinical information (including assessment of treatment response) was recorded in the corresponding medical records of the investigator, obtained as part of routine care, together with responses to the various questionnaires administered to the patient and their primary caregiver (Table 1). All questionnaires were filled in baseline, 3–4 months later (at the time of first tumor evaluation) and following the first and second disease progression. In order to evaluate family functionality, the validated Spanish version of the APGAR questionnaire was used [14]. The Family APGAR was developed in 1978 [15], and it is a 5-item questionnaire (with each item rates on a 3-point scale) measuring five constructs (adaptability, partnership, growth, affection, and resolve). The validated Spanish version of Duke-UNC-11 scale was used to measure each individual’s perception of the degree and type of social support available/received [16]. The caregiver burden in this study was assessed using the 22-item Zarit scale, which presents 22 items expressed as statements on how people who take care of a patient feel: the caregiver must select the statement that best suits how they feel. It uses a 5-point scale from 0 (never) to 4 (almost always), with a higher score indicating a higher burden. This scale has been validated in Spain by Martin et al. [17]. To assess the impact of the disease on the economy of patients and their caregivers, an ad-hoc designed questionnaire based on other previously published [18, 19] was used.

**Table 1:** Psychosocial and economic status assessment questionnaires (see these questionnaires on the “Additional files”)

Order	Patient	Caregiver
1	Family impact scale: APGAR^a^	Caregiver economic impact scale
2	Relationship impact scale	Caregiver burden scale: ZARIT^c^
3	Socio-affective impact scale (perceived support): DUKE-UNC^b^	
4	Patient economic impact scale	

^a^Score: 7–10 functional; 4–6 moderately dysfunctional; 0–3 severely dysfunctional

^b^Score: ≥ 32, normal support; < 32, low support

^c^Score: ≤ 46, no burden; > 46 and < 56, slight burden; ≥ 56: intense burden

Clinical procedures for tumor evaluation and assessment of the response to treatment were conducted in accordance with the site’s usual practice. Patients were followed until the patient’s death was documented or up to a maximum of 24 months after the end of treatment.

### Statistical analysis

Approximately 1250 patients were planned to be included. With this size, there was an 80% possibility of detecting as significant (*p* < 0.05) effect sizes less than 0.19 between men and women. The effect size serves to make the differences or relationships between different variables with different values comparable, thereby allowing us to measure the strength of the relationship between two variables. According to the Cohen classification, which defined Cohen’s d [20] for quantitative variables, a difference is considered to be large when the effect size is greater than 0.8, medium if it is greater than 0.5 and small if it is greater than 0.2 [21]. In this study, 0.19 was taken as a reference, which should enable us to detect small to large figures as significant.

Descriptive tables of the baseline and demographic characteristics of the patients are presented, as well as the results obtained from the self-administered questionnaires. All patients’ clinical and tumor characteristics at the time of diagnosis of the metastatic advanced disease were provided.

Univariate comparisons were made between the tests applied to men and women. In the case of quantitative variables, the Student’s t-test was used. Overall survival was estimated with Kaplan–Meier method. Median survival times with 95% confidence intervals (CI) were calculated. Cox analyses were done to estimate hazard ratios (HRs) with 95% CI.

All statistical tests were two-sided. The significance level was established at a value of α = 0.05. The statistical analyses were performed using SAS version 9.4.

## Results

Between February 2015 and February 2017, 333 patients (229 men and 104 women) from 20 Spanish hospitals were included. The study was prematurely closed due to a low recruitment rate. Most of demographic and baseline patients’ characteristics were similar between female and male (Table 2), with only significant differences in the number of smokers/former-smokers (97% of male and 63% of female, *p* = 0.0001) and the proportion of married/partnered (73% of male and 49% of female, *p* = 0.0002). 217 men and 85 women received chemotherapy as first line treatment, 7 men and 19 women were treated with EGFR TKI inhibitors, 4 men and 5 women received an ALK TKI inhibitor, and 1 man and 2 women received immune checkpoint inhibitors. 78 out of 104 men and 29 out of 39 women reported second-line treatment: docetaxel was the most administered drug (20 men and 9 women), followed by nivolumab (20 men and 7 women).

**Table 2:** Baseline characteristics of patients

Parameter	Arm A		Arm B		*p* value
	(Male)		(Female)		
	(N = 229)		(*N* = 104)		
	*n*	%	*n*	%	
Age, years					*0.0010*^b^
Mean (SD)	65 (8.8)		62 (10.4)		
Median (Q1, Q3)	66 (60, 72)		61 (55, 69)		
Range	36–84		37–85		
Comorbidities					0.2809^b^
Mean (SD)	3 (2.1)		3 (2.1)		
Median (Q1, Q3)	2 (1, 4)		2 (1, 4)		
Range	1–10		1–10		
ECOG					0.1643^c^
0	91	40	29	28	
1	107	47	60	58	
2	23	9	10	9	
3	2	1	1	1	
Missing	6	3	4	4	
*Smoking habits*					< *0.0001*^d^
Smoker/ex-smoker	222	97	66	63	
Non-smoker	7	3	38	37	
*Marital status*					*0.0002*^c^
Single	25	11	19	18	
* Married/Partnered*	168	73	51	49	
* Widowed*	9	4	13	13	
Divorced	4	2	6	6	
Missing	23	10	15	14	
Educational level					*0.0098*^c^
No education	25	11	9	9	
Basic	118	52	37	35	
Higher	31	13	26	25	
Missing	55	24	32	31	
Histology					*0.0082*^c^
Adenocarcinoma	158	69	87	84	
Squamous cell	44	19	8	8	
Others	27	12	8	8	
Mutations^a^					
EGFR+	11	5	21	20	
ALK+	4	2	5	5	
BRAF+	1	1	1	1	

Italic values are statistically significant

^a^EGFR mutation status was not determined in 67 men (29%) and 8 (8%) women; ALK mutation status was not determined in 103 men (45%) and 26 (25%) women; BRAF mutation status was not determined in 200 men (87%) and 93 (90%) women

^b^Wilcoxon signed-rank test

^c^Fisher’s exact test

^d^Chi-squared test

**Fig. 1:**
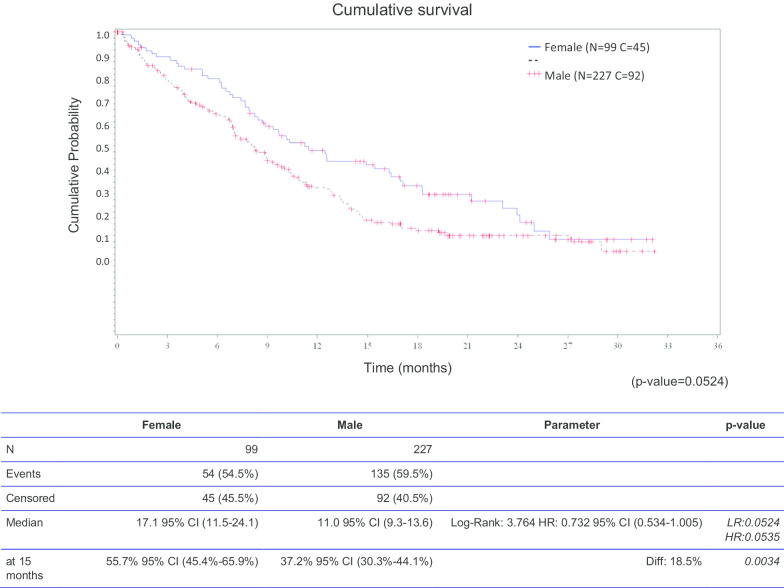
Survival by sex

The 15-monhts overall survival (OS) was 56% (95% CI 45.4–65.9%) and 37% (95% CI 30.3–44.1%) for female and male, respectively, *p* = 0.0034. The median OS was also longer in women but did not reach statistical significance (17.1 vs 11.0 months, HR 0.732 (95% CI 0.534–1.005), *p* = 0.0524) (Fig. 1).

Main baseline responses to the study questionnaires by patients and their caregivers are summarized in Tables 3 and 4. Most families were considered functional (high score in APGAR questionnaire: 83% of men and women) and, those who had a partner said their relationship had not changed (69% of men and 61% of women) or even had improved (27% of men and 39% of women). 77% of men (177/229) and 82% of women (85/104) thought their social support was normal. According to the patients’ economic questionnaire, 132 of the 195 men and 43 of the 96 women who answered the questionnaire, were retired at the beginning of the study. Around a quarter of interviewed people said their economic situation was a little worse than before suffering the disease, without remarkable differences by sex.

**Table 3:** Baseline responses to the study questionnaires: patients

Parameter	Arm A		Arm B		*p* value^c^
	(Male)		(Female)		
	(*N* = 229)		(*N* = 104)		
	*n*	%	*n*	%	
Family functionality (APGAR)					0.6961
Functional	190	83	86	83	
Moderately dysfunctional	10	4	5	5	
Severely dysfunctional	2	1	2	2	
Missing	27	12	11	10	
Partner relationship^a^					0.3235
n	137	–	46	–	
Improved	37	27	18	39	
Worsened	3	2	0	0	
Not changed	94	69	28	61	
Missing	3	2	0	0	
Social support (DUKE)					0.5372
Normal	177	77	85	82	
Low	7	3	5	5	
Missing	45	20	14	13	
Economic situation^b^					0.3476
Better	0	0	1	1	
The same	121	53	49	47	
A little bit worse	55	24	29	28	
Much worse	18	8	10	10	
Missing	35	15	15	14	

^a^Score obtained for the question “How has your relationship with your partner changed during the last month?” This item only applies to patients who have a partner (134/157 men who answered that question and 46/63 women who answered that question)

^b^Score obtained for the question “Has the disease you suffer from affected your household finances in any way?” This item only applies to patients who have a partner (134/157 men who answered that question and 46/63 women who answered that question)

^c^Fisher’s exact test

**Table 4:** Baseline responses to the study questionnaires: patients’ caregivers

Parameter	Arm A		Arm B	
	(Male patients)		(Female patients)	
	(*N* = 229)		(*N* = 104)	
	*n*	%	*n*	%
Relationship to the patient				
Parents	23	10	25	24
Partner	141	62	45	43
Brother/sister	13	6	6	6
Other family member	1	1	2	2
No relative	6	2	5	5
Missing	45	19	21	20
Work situation of the relative^a^				
Retired	55	24	26	25
Self-employed	10	4	6	6
Employed	39	17	35	33
Unemployment benefits	8	4	4	4
Home-maker	44	19	2	2
No income	10	4	4	4
Missing	63	28	27	26
Changes in working hours^b^				
No	38	17	25	24
Change in the schedule	17	7	12	12
Reduced	0	0	5	4
Stop working	8	4	3	3
Missing	166	72	59	57
Caregiving burden (Zarit scale)^c^				
No burden	116	51	54	52
Slight burden	19	8	7	7
Intense burden	10	4	4	4
Missing	84	37	39	37

^a^This question applies only to family members

^b^This question applies only to those not hired by the patient

^c^Score: ≤ 46 no burden; 47–55 slight burden; ≥ 56 intense burden

Statistically significant differences were found between both groups regarding the caregiver’s relationship to the patient, with more parents being the caregiver in females than in males (*p* < 0.0001), and the caregiver’s employment situation (more employed caregivers for female patients, *p* < 0.0001) (Table 4). 60% of caregivers of male patients (38/63) and 76% of caregivers of female who answered the economic questionnaire and who were working, had not changed their working hours. More than 80% of caregivers of both sexes who answered the Zarit questionnaire, considered that taking care of their relative did not pose a significant burden (Table 4).

Due to the relevant loss of patients during the follow-up, it was difficult to find differences by sex in the responses to the different questionnaires. According to the family APGAR questionnaire, most families remained functional across the study (Additional file 1: Online Resource 1): mean functionality levels were similar in men and women over time. The relationship with the partner did not change or improved during the course of the disease in a vast majority of men and women. The social support of the patient (Duke-UNC-11 scale) remained normal throughout the study, without appreciable differences between mean social support scores in both sexes (Additional file 2: Online Resource 2). The vast majority of patients considered the disease did not affect their household finances across the study, although a quarter of interviewed patients reported a minimum negative economic impact after lung cancer diagnosis, without remarkable differences by sex. No remarkable differences were found between the mean caregiver’s burden (Zarit scale) across the study by sex (Additional file 3: Online Resource 3), nor in the economic evaluations.

## Discussion

The Association for Lung Cancer Research in Women (ICAPEM) published a consensus paper in 2017 where a detailed analysis of the sex perspective in lung cancer was done [8]. One of the main conclusions was that there was a lack of information about evaluating social, economic and emotional impact on women with lung cancer and their caregivers/relatives. This study was set up to doing a first approach to this issue.

To the best of our knowledge, this is the first longitudinal study to investigate the impact of advanced lung cancer on patients and their caregivers from a sex perspective. As expected [22, 23] there were more smokers/ex-smokers men than women (38 out of 104 diagnosed women have never smoked). Also, there were more men than women with a partner. As reported in previous studies [24–27], median survival to 15 months and overall survival were longer in women than in men. Several reasons have been proposed to explain the improved survival of women with NSCLC, such as smoking history, comorbidities, different driver mutation pattern or differences in histology. In this study women were more likely than men to have adenocarcinoma. While some studies showed that women with adenocarcinoma had better outcomes than women with squamous cell carcinoma [28, 29], others have seen that women had better disease-free survival and OS than men regardless of stage, smoking status and histology [7, 30]. Therefore, at present, the reasons why women with NSCLC live significantly longer than men remain elusive [8].

The presence of no-relative caregivers was anecdotic in both groups, as has been reported in most of studies [31–33]. More men than women had their partners as caregivers, and more women than men had their parents as caregivers. In most of studies, partners were the most frequent family caregivers of cancer patients [13, 34, 35], mainly spouses, probably due to the social role that women still adopt. In a similar way, more family caregivers of female patients were employed, while the percentage of family caregivers of female patients being home-maker was anecdotic.

A large majority of caregivers of both sexes reported that taking care of their relative did not pose a significant baseline burden. Findings from previous studies in this area pointed that caregivers of cancer patients experienced a high level of burden [13, 36–38]. This apparent discrepancy might be due to the interpretation of the Zarit Burden Inventory in Spain [39]. This questionnaire uses a 5-point scale from 0 (never) to 4 (almost always), with a higher score indicating a higher burden. In Spain, a score ≤ 46 is considered as “No burden”, 47–55 “light burden” and ≥ 56 “Intense burden”. As stated by Ribé et al. [40], those cut points probably derive from the results of the scale validation study in Spain, carried out in a psychogeriatric centre, without considering whether it was useful and valid in any group of caregivers regardless of the characteristics of the person they care for. However, other studies consider a score of 24 as the cut-off score to identify caregivers with possible mental distress who were in need of further assessment and continued intervention, following the interpretation of Schreiner et al. [41].

No differences by sex were found throughout the study in family functionality, nor perception of social support, but around a quarter of interviewed patients said their economic situation was a little worse after the lung cancer diagnosis, without remarkable differences by sex. In addition, no remarkable differences were found between the mean caregiver’s burden across the study by sex. Nevertheless, the high loss of patients and caregivers’ responses to the questionnaires during the study, limited the statistical power for detecting significant changes/differences in outcomes.

Other limitations of this study and directions for future research should be noted. First, as it was not possible to reach the planned sample size, the statistical power for detecting significant correlates of study outcomes is limited: indeed, with the final sample size we reached, our study only have a power of 0.44 to detect as significant (*p* < 0.05) an OR of 1.4 (or its equivalent effect size of 0.19). Therefore, these results should be interpreted in an exploratory way to raise hypotheses that should be addressed in subsequent studies. Second, as stated above, the longitudinal design of the study led to the loss of a significant number of patients and caregivers who responded to the questionnaires in subsequent visits. Some of those could be due to a natural selection of patients with a better prognosis and, therefore, with a longer survival; in addition, we cannot rule out that some patients with a worse prognosis died before the first evaluation, which was performed 3–4 months after inclusion, at the time of the first tumor evaluation (usual practice). It is highly recommended to do a thorough monitoring of this kind of studies and to use recapture strategies such as telephone calls after each visit. Third, Spain has a National Healthcare System that covers the main costs of diseases, including treatments (pharmacological and others), hospitalisation, outpatient visits and tests. In addition, the low percentage of people who acknowledged that their economic situation had worsened due to the disease, may also be related to the Mediterranean culture, where it is not appropriate to talk about money.

## Conclusion

Despite its limitations, the present study provides a preliminary insight into sex-related characteristics in the management of advanced NSCLC and its impact on the emotional, social and economic burden of patients and their caregivers, and recall the high priority of researching in cancer from a sex perspective.

## Supplementary information

**Additional file 1.** Online Resource 1: Family functionality (APGAR questionnaire) by sex over time.

**Additional file 2.** Online Resource 2: Social support (Duke-UNC-11 scale) by sex over time.

**Additional file 3.** Online Resource 3: Caregiving burden (Zarit scale) by patient sex over time.

## Data Availability

The datasets during and/or analysed during the current study available from the corresponding author on reasonable request.

## Abbreviations

APGAR: Relationship impact scale
CI: Confidence interval
DUKE-UNC: Scale, economic impact in patients and caregiver
ECOG: Eastern Cooperative Oncology Group performance status
HR: Hazard ratios
ICAPEM: Spanish Association for Lung Cancer Research in Women
INE: Spanish Statistics National Institute
mNSCLC: Metastatic non-small cell lung cancer
NSCLC: Non-small cell lung cancer
OS: Overall survival
