# Supplementary material for: Assessment of the psychosocial and economic impact according to sex in non-small cell lung cancer patients: an exploratory longitudinal study
Source: BMC Psychol. 2020 Nov 23;8:123. doi: 10.1186/s40359-020-00489-z (PMC7685640; doi:10.1186/s40359-020-00489-z)
Supplement: Supplementary file 2 — Additional file 2. Online Resource 2: Social support (Duke-UNC-11 scale) by sex over time. [file 40359_2020_489_MOESM2_ESM.pdf]

Assessment of the psychosocial and economic impact according to sex in non-small cell lung cancer patients: an exploratory longitudinal study

Nuria Viñolas N, Rosario Garcia-Campelo, Margarita Majem, Enric Carcereny, Dolores Isla, José Luis Gonzalez-LarribaL, Juan Coves, Javier De-Castro, Manuel Domine, Piar Lianes, Angel Artal, Jordi Remon, Enriqueta Felip, Pilar Garrido.

Corresponding author:

Nuria Viñolas

Medical Oncology Department

Hospital Clínic i Provincial de Barcelona

Carrer Villarroel, 170

08036 Barcelona, Spain

nvinolas@clinic.cat

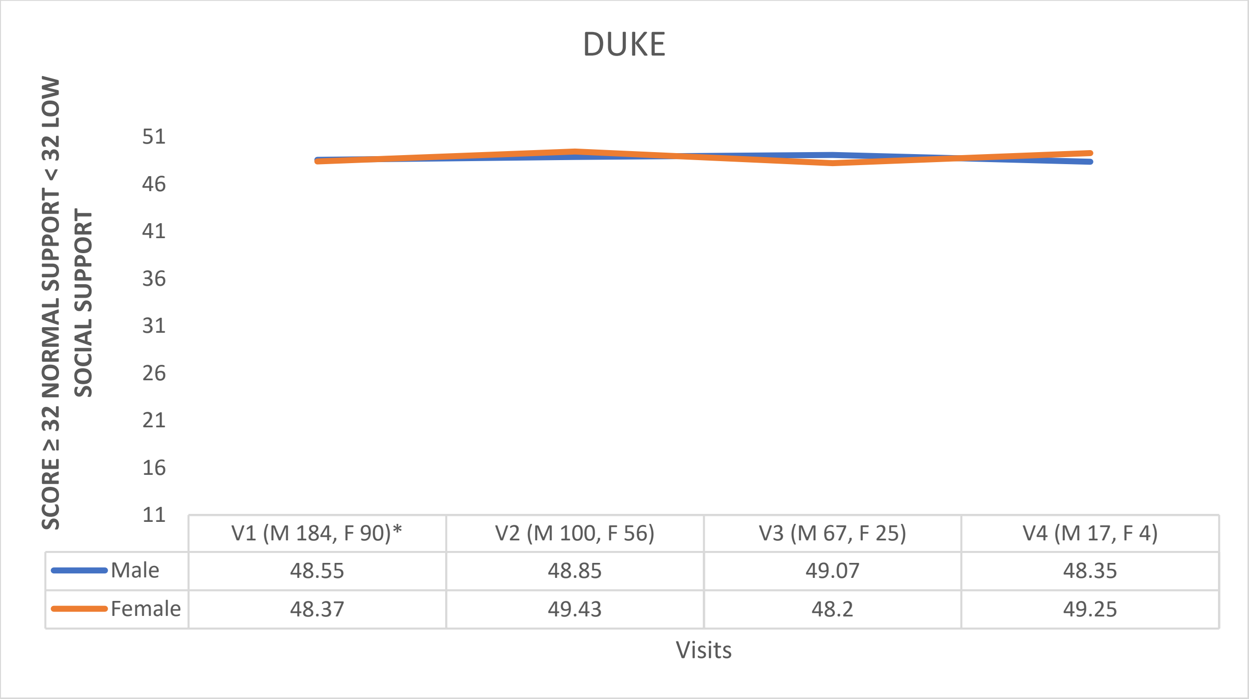

* Number of males (M) and females (F) who answered the questionnaire at each visit.
